# Diurnal Variation of Sweet Taste Recognition Thresholds Is Absent in Overweight and Obese Humans

**DOI:** 10.3390/nu10030297

**Published:** 2018-03-02

**Authors:** Keisuke Sanematsu, Yuki Nakamura, Masatoshi Nomura, Noriatsu Shigemura, Yuzo Ninomiya

**Affiliations:** 1 Section of Oral Neuroscience, Graduate School of Dental Sciences, Kyushu University, Fukuoka 812-8582, Japan; sanematu@dent.kyushu-u.ac.jp (K.S.); nakayuki@dent.niigata-u.ac.jp (Y.N.); shigemura@dent.kyushu-u.ac.jp (N.S.); 2 Division of Sensory Physiology, Research & Development Center for Taste and Odor Sensing, Kyushu University, Fukuoka 812-8582, Japan; 3 Department of Medicine and Bioregulatory Science, Graduate School of Medical Sciences, Kyushu University, Fukuoka 812-8582, Japan; nomura@med.kyushu-u.ac.jp; 4 Division of Endocrinology and Metabolism, School of Medicine, Kurume University, Fukuoka 830-0011, Japan; 5 Monell Chemical Senses Center, Philadelphia, PA 19104, USA

**Keywords:** obesity, taste, leptin, insulin resistance, human

## Abstract

Sweet taste thresholds are positively related to plasma leptin levels in normal weight humans: both show parallel diurnal variations and associations with postprandial glucose and insulin rises. Here, we tested whether this relationship also exists in overweight and obese (OW/Ob) individuals with hyperleptinemia. We tested 36 Japanese OW/Ob subjects (body mass index (BMI) > 25 kg/m^2^) for recognition thresholds for various taste stimuli at seven different time points from 8:00 a.m. to 10:00 p.m. using the staircase methodology, and measured plasma leptin, insulin, and blood glucose levels before each taste threshold measurement. We also used the homeostatic model assessment of insulin resistance (HOMA-IR) to evaluate insulin resistance. The results demonstrated that, unlike normal weight subjects, OW/Ob subjects showed no significant diurnal variations in the recognition thresholds for sweet stimuli but exhibited negative associations between the diurnal variations of both leptin and sweet recognition thresholds and the HOMA-IR scores. These findings suggest that in OW/Ob subjects, the basal leptin levels (~20 ng/mL) may already exceed leptin’s effective concentration for the modulation of sweet sensitivity and that this leptin resistance-based attenuation of the diurnal variations of the sweet taste recognition thresholds may also be indirectly linked to insulin resistance in OW/Ob subjects.

## 1. Introduction

Leptin is a hormone that regulates food intake, energy expenditure, and body weight mainly through the activation of the hypothalamic functional leptin receptor (Ob-Rb) [1,2]. Our previous studies in mice showed that the peripheral taste organ is also a target of leptin that acts on taste receptor cells via Ob-Rb receptors expressed in these cells [3,4]. Leptin specifically inhibits the responses to sweet substances without affecting the responses to sour, salty, and bitter substances in the chorda timpani (CT) nerve innervating the anterior two-thirds of the tongue in lean mice. Such selective sweet response inhibition by leptin was not observed in leptin receptor-deficient obese diabetic db/db mice [3,4]. This suggests that leptin may act as a sweet taste inhibitor involved in the regulation of food intake.

Our subsequent human study with normal weight (NW) subjects demonstrated that the recognition thresholds for sweet compounds exhibit circadian variations which parallel circulating leptin levels. That is, both sweet recognition thresholds and leptin levels begin rising before noon and peak in the night [5]. When leptin level variations were phase-shifted by altering the number of meals per day, the diurnal variation of the thresholds for sweet compounds shifted in parallel. This synchronization of the diurnal variation in leptin levels with the sweet taste recognition thresholds suggests a mechanistic connection between these two variables. In mice, consistent with this tendency, leptin’s ability to suppress sweet taste responses saturates at around 15 ng/mL [3,6,7]. Since obese individuals generally have higher basal leptin levels compared with NW individuals [8,9], this leads to the predictions that the obese would exhibit much smaller diurnal variations of sweet taste sensitivity than NW individuals. Also, relative to NW individuals, obese individuals generally show insulin resistance, higher insulin levels, and slower postprandial glucose clearance [10]. These characteristics in obese individuals may be linked with their higher basal leptin levels because leptin may influence glucose absorption in the gastrointestinal tract [11] and insulin secretion in the pancreatic islets [12,13]. In the present study, therefore, we tested the possibility that, unlike NW individuals, obese and overweight (OW/Ob) individuals would not exhibit correlated changes in leptin levels and sweet taste sensitivity, but would show a linkage among leptin, insulin, glucose levels, and sweet taste sensitivity. To test this hypothesis, we compared the recognition thresholds for various taste stimuli, plasma leptin, insulin, and blood glucose levels at seven time points during the day in Japanese OW/Ob adults (body mass index BMI > 25 kg/m^2^). The results indicated that OW/Ob subjects showed diurnal variations in leptin levels but not in sweet taste thresholds. We also found that the diurnal variation of leptin and sweet recognition thresholds were negatively associated with the homeostatic model assessment of insulin resistance (HOMA-IR) scores in OW/Ob subjects. These results provide valuable insight into the mechanistic connection among plasma leptin levels, sweet sensitivity, and insulin resistance in OW/Ob subjects.

## 2. Materials and Methods

### 2.1. Subjects and Experimental Protocol

We studied a total of 36 OW/Ob subjects (male/female: 17/19, age: 23–67, BMI: 25.8–52.1 kg/m^2^) who joined a weight-loss program in Kyushu University hospital. The subjects were tested on the second day of the program. The inclusion criteria were: a satisfactory state of oral hygiene, non-smoking, regular work, sleep, and regular meal schedules. The purpose of the study as well as the methods and procedure were explained to the participants, and their informed consent was received. The experimental protocol was approved by the institutional review board at Kyushu University (Ref. No. 15A-4). The female subjects were tested between 3–16 days after the end of the menstrual phase in the menstrual cycle. All subjects were asked not to eat or drink anything other than water after 10:00 p.m. the evening before the experiment and to abstain from snacks and tooth paste during the day of the experiment. A blood sample was taken, and taste recognition thresholds were measured 30 min before and 1 h after each meal and at 10:00 p.m. (seven times a day). Meal times were as follows: breakfast 8:30 a.m., lunch 12:30 p.m., and dinner 5:30 p.m. The caloric values for each meal were 470 kcal, 475 kcal, and 475 kcal for breakfast, lunch, and dinner, respectively. The subjects were placed on a diet consisting of 24% fat, 60% carbohydrate, and 16% protein. In our former work [5], NW subjects received 2200 kcal when provided with three meals consisting of 29% fat, 57% carbohydrate, and 14% protein. The macronutrient compositions of meals in OW/Ob and NW subjects were as for the typical Japanese diet. 

### 2.2. Taste Recognition Threshold Measurement

Recognition thresholds were measured for sweet, salty, sour, bitter, and umami taste qualities. Test stimuli and concentrations were as follows: sucrose (0.001–0.1 M), glucose (0.001–0.56 M), sodium saccharin (0.001–1.0 mM), NaCl (0.001–1.0 M), citric acid (0.01–10.0 mM), quinine HCl (QHCl) (0.0001–0.1 mM), and monosodium glutamate (MSG) (0.1–100.0 mM). The differences in concentrations in the present study were in 0.25 log steps. All solutions were made in distilled water and used at room temperature. The subjects were also taste-tested with 1.0 mM phenylthiocarbamide (PTC), and we found that all 36 OW/Ob subjects were PTC tasters. 

The testing procedure was the staircase method [5], as follows: Each subject evaluated all seven tastants. The seven series of solutions were presented one after another in random order. Within each series, the solutions (each 2 mL) were applied to the whole tongue of each subject by a pipet in order of ascending concentration. The subjects were asked not to swallow the test solutions and to rinse their mouths between two solutions with distilled water. Each subject was requested to correctly name the tastant in each series. Once the taste of two successive concentrations was recognized successfully, the subject was given the previously unrecognized concentration (first reversal). The points at which the concentration sequence changed from decreasing concentration to increasing concentration or vice versa were designated “reversals”. The procedure was terminated after five reversals, and the threshold was calculated as the mean of the log concentration values of the last four reversals. Before the test trial, the subjects attended a pre-trial session to learn the staircase method, during which we measured the recognition thresholds as a reference for the following trial. During the test trial, we used, as an initial concentration to apply to the subjects, a concentration lower than the recognition thresholds for each taste obtained from the pre-trial session or a previous trial for time saving.

### 2.3. Assays

Blood samples were drawn for the determination of plasma leptin, insulin, and glucose. After the blood samples were centrifuged at 4 °C (3000 rpm. for 15 min), plasma samples were collected and stored at −80 °C. Plasma leptin and insulin concentrations were determined using an ELISA kit (R&D Systems) and a sensitive ELISA method (LUMIPULSE, Fujirebio Inc., Malvern, PA, USA), respectively. All samples were within the linear detection range and were analyzed in duplicate. Blood glucose levels were determined by the glucose dehydrogenase method (ACCU-CHEK; Roche Diagnostics KK, Tokyo, Japan).

### 2.4. Data Analysis

Leptin, blood glucose, insulin levels, and taste recognition thresholds, expressed as mean ± SE (standard error), were first used for statistical analyses to evaluate circadian variations. Only the taste recognition thresholds were log transformed. Then, the values from 8:00 a.m. to 10:00 p.m. were expressed as percent changes from the 8:00 a.m. values (control = 100%). Statistical significance was determined by unpaired *t-*tests. To evaluate the variations, the one-way or two-way repeated measures analysis of variance (ANOVA) was performed. The quantitative insulin sensitivity check index (QUICKI) (=1/(log fasting insulin + log fasting glucose)) and HOMA-IR (=fasting insulin × fasting glucose/405) were calculated for a rough estimation of insulin sensitivity and resistance [14,15]. For the measurement of the association between diurnal variation of plasma leptin and taste recognition thresholds with HOMA-IR, a linear regression analysis was performed.

## 3. Results

Table 1 shows plasma leptin, insulin and blood glucose levels, and taste recognition thresholds for the seven taste stimuli at 8:00 a.m. after overnight fasting in male and female OW/Ob subjects. Consistent with our previous report on NW subjects [5], a significant gender difference was observed in plasma leptin levels (*p* < 0.01), whereas no such difference was observed in plasma insulin, blood glucose, and taste recognition thresholds for any stimulus in the OW/Ob subjects (*p* > 0.05). Mean QUICKI [14] and HOMA-IR [14] of the 36 subjects indicated that they, as a whole, had decreased insulin sensitivity (QUICKI index < 0.357) [16] and insulin resistance (HOMA-IR > 2.5) [17]. 

**Figure 1:**
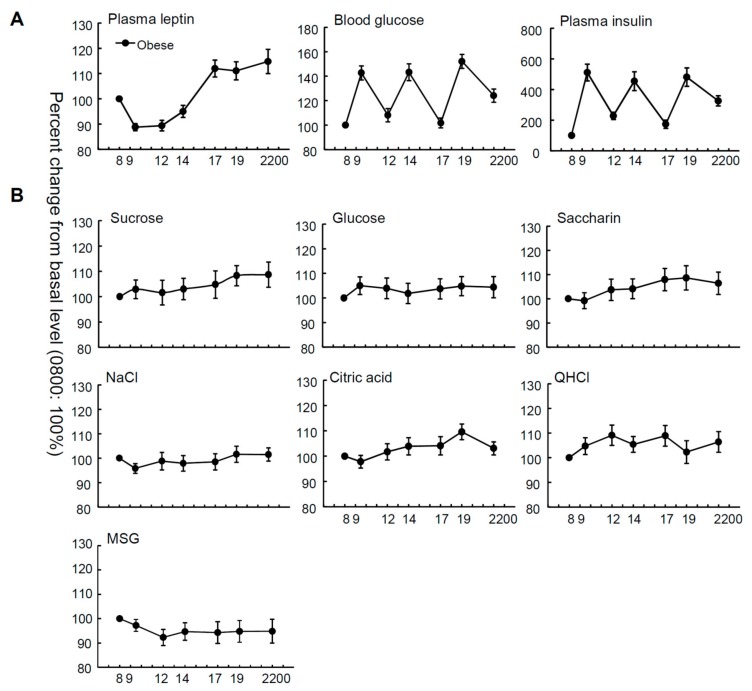
Time courses of plasma leptin, blood glucose, plasma insulin levels, and taste recognition thresholds. Mean plasma leptin, blood glucose, and plasma insulin levels (**A**); and recognition thresholds for seven taste stimuli (**B**) measured at seven different time points during the day from 8:00 a.m. to 10:00 p.m. in overweight and obese subjects (*n* = 36). The value at each point is a percentage of the value at 8:00 a.m. (control = 100%).

Figure 1 and Figure S1 show the mean relative and absolute values of plasma leptin, insulin and blood glucose levels, and taste recognition thresholds for seven stimuli at seven time points from 8:00 a.m. to 10:00 p.m. in OW/Ob subjects. The relative plasma leptin concentrations of the OW/Ob subjects showed diurnal variation, whereby the level started rising before noon and peaked in the night (Figure 1, Table 1), although significant diurnal variation was not observed in the absolute values of plasma leptin (Figure S1, Table S1). Statistical analyses indicated significant time-dependent changes in leptin, glucose, and insulin levels (Table 1, all *p* < 0.001). In contrast, with regard to the taste recognition thresholds, the time-dependent changes in the thresholds for all taste stimuli tested were not significant (Figure 1, Table 1, all *p* > 0.05), suggesting that the diurnal variations of the mean plasma leptin levels were not synchronized with those of the sweet taste recognition thresholds.

**Figure 2:**
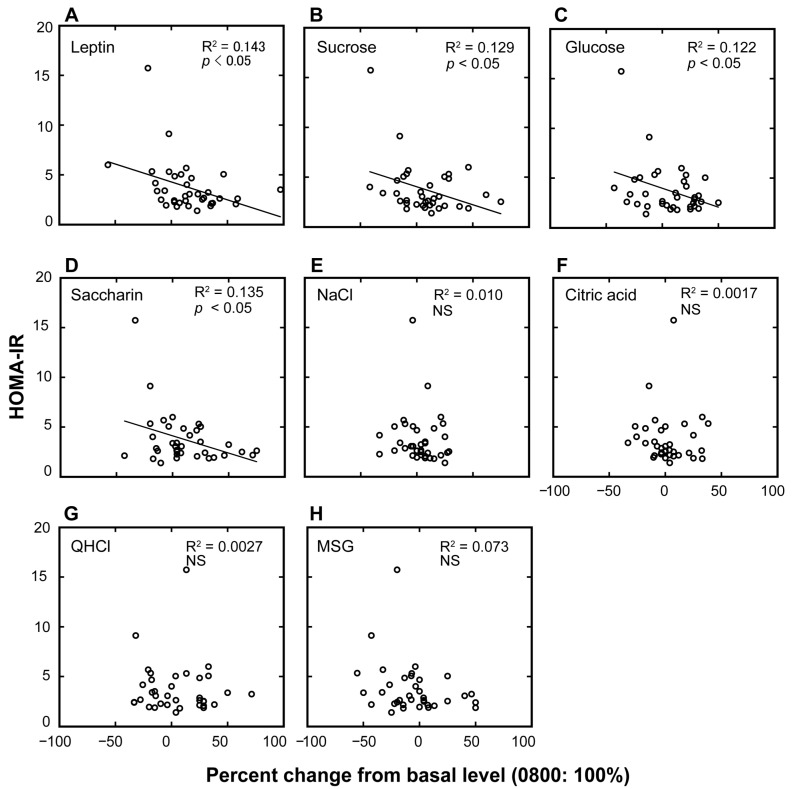
Association plots between the homeostatic model assessment of insulin resistance (HOMA-IR) values and the percent changes in plasma leptin levels and taste recognition thresholds. Association plots between HOMA-IR scores and percent changes in plasma leptin levels (**A**) and taste recognition thresholds for seven different taste stimuli (**B**–**H**) at 10:00 p.m. compared with the basal values collected at 8:00 a.m. (control = 100%) in overweight and obese individuals. Each circle represents a single individual subject. Linear regression analysis was used to obtain the coefficient of determination (*n* = 36). NS, not significant.

Next, we examined individual differences in the diurnal variations of the plasma leptin levels and taste recognition thresholds for each of the seven taste stimuli and their potential links with HOMA-IR scores (Figure 2 and Table 2). Table 2 summarizes the linear regressions between HOMA-IR scores and the magnitudes of the diurnal variations (percent increase of the value at 10:00 p.m. from that at 8:00 a.m.) of leptin and of the taste recognition thresholds. The HOMA-IR scores were negatively associated with the percent changes in plasma leptin and taste recognition thresholds for sucrose, glucose, and saccharin (*p* < 0.05).

## 4. Discussion

Our previous studies in mice showed that the administration of leptin specifically suppressed CT nerve responses to sweet compounds via Ob-Rb receptors expressed in sweet-responsive cells. This suppressive effect of leptin occurred at plasma leptin levels from around 2 ng/mL and saturates at around 15 ng/mL [3]. Blocking Ob-Rb receptors by the administration of a leptin antagonist specifically enhanced the responses to sweet compounds, and this effect was not observed in diet-induced obese mice with above 20 ng/mL of plasma leptin [6]. Consistently, the present study in humans revealed that the diurnal variation of the mean recognition thresholds for sweet substances previously found in NW subjects [5] was not evident in OW/Ob subjects. This may be due to the higher basal leptin level in OW/Ob individuals (morning values ~20 ng/mL) compared with NW individuals. That is, the potential effect of leptin on sweet taste recognition thresholds may already be at maximum in the morning, and any increase of leptin levels cannot therefore influence the taste recognition thresholds. 

It is generally known that the principal taste receptor for sugars and other sweeteners is a heterodimer of taste receptor type 1 members 2 and 3 (T1R2/T1R3), because mice genetically lacking T1R2/T1R3 are shown to display severely diminished neural and behavioral taste responses to sugars, artificial sweeteners, and some amino acids [18,19]. Recently, there has also been growing evidence suggesting that there may be a T1R-independent sugar-specific reception pathway formed by glucose transporters, an adenosine triphosphate (ATP)-gated K^+^ (K_ATP_) channel known as a metabolic sensor [20,21], and other downstream transduction molecules. Our recent study demonstrated that leptin may activate the K_ATP_ channel in T1R3-expressing taste cells [7] and lead to a reduction of the excitability of sweet-responsive taste cells. If this is also the case in the human taste system, it is possible that leptin and its resistance may more preferentially affect sugar-specific pathways containing K_ATP_ channels than the T1R2/T1R3 pathway sensitive to all sweet compounds including artificial sweeteners. However, in the present study, OW/Ob subjects showed no significant diurnal variation of the mean recognition thresholds, not only for sugars but also for saccharin. This may suggest no distinguishable differences in leptin effects on the two different potential sweet reception pathways. In mice, K_ATP_ channels were shown to be expressed in about 80% of T1R3-expressing taste cells, suggesting a substantial overlap between the two sweet reception pathways in the same sweet-responsive cells [7,20]. Therefore, the activation of K_ATP_ channels by leptin may lead to a reduction of the whole cell excitability of sweet taste cells, followed by the inhibition of taste responses to all sweet compounds, including saccharin, which would be solely detected by the T1R2/T1R3 pathway. However, further studies are needed to clarify whether leptin would more preferentially influence the sugar-specific pathway. 

In our study, the total energy intake was reduced to promote loss of weight in OW/Ob subjects, and this may affect the diurnal variations of leptin and taste recognition thresholds. In previous studies, diet-induced weight loss in a long-term trial (4−6 months) produced a coordinate decrease in plasma leptin levels and an increase in the amplitude of the 24 h leptin signal [22,23]. In our study, the basal leptin level in OW/Ob subjects was higher than that in NW subjects, although the average age and total calorie intake were not matched between the two groups (Table S2). The amplitude of the diurnal leptin levels in OW/Ob subjects was slightly decreased but not lost. Therefore, these effects observed in previous studies [22,23] were not observed in OW/Ob subjects in our study because of the short period of calorie reduction.

Although we found no direct correlation between leptin levels and sweet taste recognition thresholds in OW/Ob subjects, we did observe that the magnitudes of the diurnal variations of leptin and of the recognition thresholds for sweet compounds (sucrose, glucose, and saccharin) were significantly and negatively associated with the insulin resistance HOMA-IR values, calculated on the basis of fasting insulin and glucose levels. In relation to this association, recent studies demonstrated that gut enteroendocrine cells, like sweet taste cells, express T1R2/T1R3 in both rodents and humans [24,25,26,27]. The activation of the sweet receptors by sugars elicits the release of glucagon-like peptide-1 which leads to an increase in the expression of the Na^+^/glucose co-transporter, followed by increased glucose absorption in enterocytes in mice [24,25]. Also, gut leptin induces the inhibition of sugar absorption in rat intestine [11,28,29], and leptin’s effect on the absorption of glucose saturates at around 16 ng/mL [11]. Leptin affects the sweet taste responses of the mouse enteroendocrine cell line STC-1 cells through the activation of the leptin receptor and the K_ATP_ channels expressed in these cells, with a maximum level at 20 ng/mL [30]. If this is also the case in humans, OW/Ob subjects in the present study who have high leptin levels (~20 ng/mL) might show no further regulation (inhibition) by leptin on both sweet sensitivity and glucose absorption in the gut, which may lead to an increase of glucose absorption. However, it is also possible that these associations may be secondary and not causal or relevant to the leptin levels. Further studies are required to elucidate the mechanisms underlying these associations.

As for leptin’s action on pancreatic islets, previous studies in rodents provided controversial results showing decrease [31,32], no effect [33], or increase [34] of insulin secretion induced by leptin below 50 ng/mL. However, in human islets, human leptin below 50 ng/mL produced no effect, and only higher concentrations (≥100 ng/mL) of leptin inhibited insulin secretion [12,13]. In in vivo studies, plasma leptin levels at concentrations below 80 ng/mL in non-obese and obese adults [35,36] were positively correlated with insulin concentration. In the present study, 35 out of 36 OW/Ob subjects showed leptin levels below 50 ng/mL. Within this concentration range, leptin levels may still be positively related to basal insulin levels. Collectively, this difference in the effective concentration ranges of leptin between taste cells/gut enteroendocrine cells and pancreatic beta cells may be one of the factors that may lead to relatively higher plasma insulin levels and concomitant increases in the HOMA-IR scores in OW/Ob subjects with higher leptin levels (15–80 ng/mL) (Table S2). This may underlie the negative relationships between the diurnal variations of both leptin and sweet sensitivity and the HOMA-IR scores in OW/Ob subjects.

Previous studies suggest a potential link between leptin resistance and insulin resistance. In fact, similar to the manifestation of insulin resistance, the majority of obese patients and diet-induced obese mice show leptin resistance [6,37,38,39,40]. Endoplasmic reticulum stress decreases or completely suppresses leptin signaling in obese humans with type 2 diabetes, suggesting that the negative effect of the endoplasmic reticulum on leptin sensitivity may also affect insulin resistance [41,42]. Localized increases of suppressor of cytokine signaling 3 (SOCS3), known as an inhibitor of leptin and insulin signaling specifically in the hypothalamus, may lead to leptin and insulin resistance [43,44,45]. Therefore, negative associations between the magnitude of the diurnal variations in sweet taste recognition thresholds and insulin resistance HOMA-IR scores in the present study may be due to the negative effect on sweet sensitivity as a result of leptin resistance, which is associated with insulin resistance. 

Resistance to insulin or leptin does not develop uniformly throughout the organism. These types of resistance suppress intracellular signaling pathways in particular cell types and organs, whereas the action of insulin and leptin is maintained in others [46,47,48]. Although there are many reports in relation to the mechanisms underlying the effects of leptin and insulin [49,50], future studies are needed to provide a well-defined mechanism responsible for the development of the resistance to insulin and leptin and the absence of diurnal variations of sweet sensitivity.

## 5. Conclusions

In summary, the present study showed that, unlike NW individuals [5], OW/Ob individuals do not exhibit circadian variation in the recognition thresholds for sweet compounds. However, in these OW/Ob individuals, the magnitudes of the diurnal variation for plasma leptin and sweet taste recognition thresholds were negatively associated with insulin resistance HOMA-IR scores. This leptin-based attenuation of the diurnal variations in sweet taste recognition thresholds may also be indirectly linked with the development of insulin resistance in OW/Ob subjects.

## Figures and Tables

**Table 1:** Comparison of plasma leptin, blood glucose, plasma insulin, values for insulin resistance/sensitivity (HOMA-IR/QUICKI), BMI, and taste recognition thresholds for seven taste stimuli between male and female subjects and results of the statistical analyses of time-dependent changes of these values. BMI: body mass index; HOMA-IR: homeostatic model assessment of insulin resistance; QUICKI: quantitative insulin sensitivity check index.

	Male (*n* = 17)	Female (*n* = 19)	*t*-Test	All Subjects (*n* = 36)	One-Way Repeated Measures ANOVA	
			*p*		*F*	*p*
Leptin (ng/mL)	12.3 ± 1.6	26.8 ± 4.4	<0.01	20.2 ± 2.7	(6, 245) = 16.18	<0.001
Blood glucose (mg/dL)	97.7 ± 5.9	105.4 ± 8.4	NS	102.3 ± 5.2	(6, 245) = 18.81	<0.001
Insulin (μIU/mL)	13.6 ± 1.7	14.5 ± 1.9	NS	14.1 ± 1.2	(6, 245) = 15.43	<0.001
HOMA-IR	3.15 ± 0.46	4.18 ± 0.73	NS	3.69 ± 0.45		
QUICKI	0.32 ± 0.005	0.32 ± 0.0047	NS	0.32 ± 0.0036		
BMI (kg/m^2^)	30.8 ± 1.1	34.7 ± 1.9	NS	32.9 ± 1.1		
**Taste Recognition Thresholds (mM)**						
Sucrose	30.7± 3.3	23.4 ± 3.6	NS	26.8 ± 2.5	(6, 243) = 0.66	NS
Glucose	131.4 ± 10.9	96.8 ± 17.1	NS	113.1 ± 10.6	(6, 244) = 0.29	NS
Saccharin	0.12 ± 0.015	0.11 ± 0.020	NS	0.11 ± 0.011	(6, 237) = 0.99	NS
NaCl	31.9 ± 3.8	27.2 ± 4.8	NS	29.4 ± 3.0	(6, 237) = 0.66	NS
citric acid	0.56 ± 0.05	0.53 ± 0.08	NS	0.55 ± 0.05	(6, 245) = 2.18	NS
QHCl	0.015 ± 0.003	0.015 ± 0.002	NS	0.015 ± 0.002	(6, 245) = 0.99	NS
MSG	13.6 ± 2.8	9.9 ± 2.2	NS	11.5 ± 1.8	(6, 231) = 0.53	NS

NS: not significant.

**Table 2:** Relationships between HOMA-IR values and (a) percent changes in plasma leptin levels and taste recognition thresholds for (b) sucrose, (c) glucose, (d) saccharin, (e) NaCl, (f) citric acid, (g) QHCl, and (h) MSG at 10:00 p.m. compared with the basal values collected at 8:00 a.m. (control = 100%) in all the individuals tested.

HOMA-IR			
	β	Adjusted *R*^2^	*p*
a. plasma leptin	−0.379	0.12	<0.05
b. sucrose	−0.360	0.10	<0.05
c. glucose	−0.350	0.10	<0.05
d. saccharin	−0.367	0.11	<0.05
e. NaCl	−0.101	−0.02	NS
f. citric acid	−0.041	−0.03	NS
g. QHCl	−0.005	−0.03	NS
h. MSG	−0.269	0.05	NS

Linear regression analysis was used to assess the relationships; β: standardized partial regression coefficient, NS: not significant, *n* = 36; QHCl: quinine HCl; MSG: monosodium glutamate.

## Supplementary Materials

The following are available online at http://www.mdpi.com/2072-6643/10/3/297/s1, Figure S1: Time courses of plasma, leptin, blood glucose, plasma insulin levels, and taste recognition thresholds, Table S1: Statistical analyses of time-dependent changes in leptin levels and recognition thresholds, Table S2: Plasma leptin, blood glucose, insulin levels, and taste recognition thresholds in non-obese and overweight/obese subjects.
