# Supplementary material for: Diurnal Variation of Sweet Taste Recognition Thresholds Is Absent in Overweight and Obese Humans
Source: Nutrients. 2018 Mar 2;10(3):297. doi: 10.3390/nu10030297 (PMC5872715; doi:10.3390/nu10030297)
Supplement: Supplementary file 1 [file nutrients-10-00297-s001.pdf]

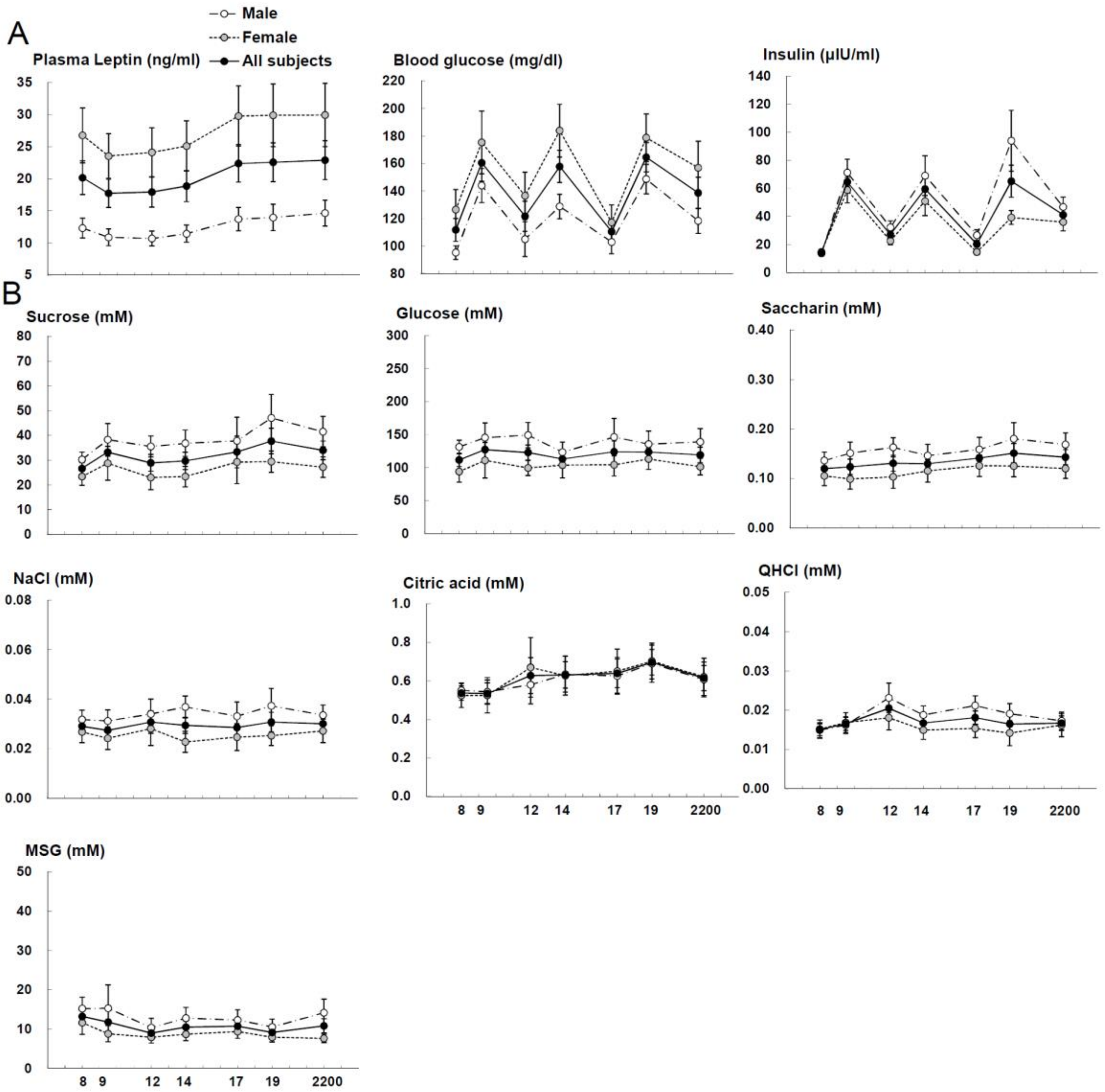

Figure S1. Time courses of plasma, leptin, blood glucose, plasma insulin levels, and taste recognition thresholds. Mean plasma leptin, blood glucose and plasma insulin levels (A), and recognition thresholds for seven taste stimuli (B) measured at seven different time points during the day from ~~0~~8:00 a.m. to 10~~22~~:00 a.m. in over-weight and obese subjects (n = 36). The values at each point are absolute values.

Table S1. Statistical analyses of time-dependent changes in leptin levels and recognition thresholds-.

	One-Way Repeated-Measures ANOVA						Two-Way Repeated-Measures ANOVA			
	**Male (n=17)**		**Female (n=19)**		**All subjects (n=36)**		Effect of gender		Gender-Time change interaction	
	F	***Pp***	***F***	***Pp***	***F***	***pP***	***F***	***pP***	***F***	***Pp***
Leptin (ng/ml)	(6, 112) = 0.95	NS	(6, 126) = 0.42	NS	(6, 245) = 0.70	NS	(1, 34) = 9.54	<0.01	(6, 204) = 1.52	NS
Blood glucose (mg/dl)	(6, 112) = 4.42	<0.001	(6, 126) = 2.30	<0.05	(6, 245) = 4.77	<0.001	(1, 34) = 3.01	NS	(6, 204) = 1.63	NS
Insulin (µIU/ml)	(6, 112) = 6.77	<0.001	(6, 126) = 7.89	<0.001	(6, 245) = 11.6	<0.001	(1, 34) = 4.03	NS	(6, 204) = 3.27	<0.01
Taste recognition thresholds (mM)										
Sucrose	(6, 111) = 0.59	NS	(6, 125) = 0.29	NS	(6, 243) = 0.73	NS	(1, 34) = 3.51	NS	(6, 202) = 0.35	NS
Glucose	(6, 112) = 0.22	NS	(6, 125) = 0.14	NS	(6, 244) = 0.20	NS	(1, 34) = 3.60	NS	(6, 203) = 0.26	NS
Saccharin	(6, 109) = 0.39	NS	(6, 121) = 0.26	NS	(6, 237) = 0.49	NS	(1, 34) = 3.24	NS	(6, 196) = 0.32	NS
NaCl	(6, 109) = 0.16	NS	(6, 121) = 0.14	NS	(6, 237) = 0.12	NS	(1, 34) = 1.70	NS	(6, 196) = 0.65	NS
Citric acid	(6, 112) = 0.37	NS	(6, 126) = 0.43	NS	(6, 245) = 0.74	NS	(1, 34) = 0.012	NS	(6, 204) = 0.21	NS
QHCl	(6, 112) = 1.25	NS	(6, 126) = 0.24	NS	(6, 245) = 0.84	NS	(1, 34) = 0.92	NS	(6, 204) = 1.20	NS
MSG	(6, 105) = 0.40	NS	(6, 119) = 0.56	NS	(6, 231) = 0.65	NS	(1, 34) = 1.01	NS	(6, 190) = 1.91	NS

The table is based on data shown in Figure S1; NS, not significant

Table S2. Plasma leptin, blood glucose, insulin levels, and taste recognition thresholds in non-obese and over-weight/obese subjects.

	**Non-obese***	**Over-weight/obese**	~~*P*~~*p*
Number of subjects	47	36	
Age	25.04 ± 0.39	42.89 ± 2.23	<0.001
Leptin (ng/ml)	6.43 ± 0.74	20.2 ± 2.7	<0.001
Blood glucose (mg/dl)	96.3 ± 2.0	102.3 ± 5.2	NS
Insulin (μIU/mL)	6.7 ± 0.67	14.1 ± 1.2	<0.001
HOMA-IR	1.46 ± 0.16	3.69 ± 0.45	<0.001
QUICKI	0.38 ± 0.008	0.32 ± 0.0036	<0.001
Taste recognition thresholds (mM)			
Sucrose	23.0 ± 2.5	26.8 ± 2.5	NS
Glucose	95.3 ± 7.7	113.1 ± 10.6	NS
Saccharin	0.087 ± 0.011	0.11 ± 0.011	NS
NaCl	24.8 ± 2.7	29.4 ± 3.0	NS
Citric acid	0.47 ± 0.06	0.55 ± 0.05	NS
QHCl	0.012 ± 0.001	0.015 ± 0.002	NS
MSG	4.2 ± 0.6	11.5 ± 1.8	<0.001

Data show mean ± S.E. at ~~0~~8:00 a.m.~~h~~ after overnight fasting. ~~*P*~~*p* < 0.001 by unpaired *t* test between values for non-obese and obese subjects. NS, not significant. * Nakamura *et al*., *Diabetes* **2008**, *57*, 2661–5
